# Supplementary material for: Thermally Controlled State Switches for Engineered Macrophages
Source: ACS Synth Biol. 2025 Oct 11;14(11):4304–13. doi: 10.1021/acssynbio.5c00395 (PMC12645579; doi:10.1021/acssynbio.5c00395)
Supplement: Supplementary file 3 [file sb5c00395_si_003.pdf]

Supporting Information

Thermally controlled state switches for engineered macrophages

Ann Liu[1]*, Abdullah S. Farooq[1]*, Mohamad H. Abedi[1], Ernesto Criado-Hidalgo[2,3], Cameron A. B. Smith[2], Di Wu[2,#], Mikhail G. Shapiro[2,3,4,#]

[1]Division of Biology and Biological Engineering, California Institute of Technology; Pasadena, CA 91125, USA
[2]Division of Chemistry and Chemical Engineering, California Institute of Technology; Pasadena, CA 91125, USA
[3]Andrew and Peggy Cherng Department of Medical Engineering, California Institute of Technology; Pasadena, CA 91125, USA
[4]Howard Hughes Medical Institute; Pasadena, CA 91125, USA
[*]Contributed equally
[#]Corresponding authors: dwwu@caltech.edu, mikhail@caltech.edu

Contents: Figures S1-12

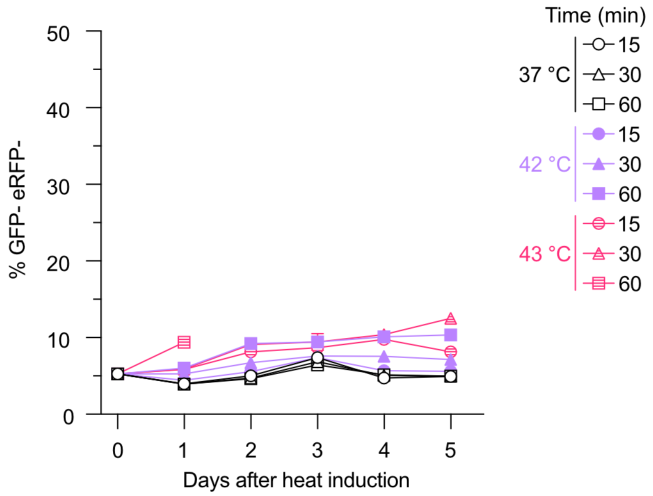

Figure S1. Double negative population of RAW-togFP cells after heating.
Percentage of GFP- and eRFP- RAW-togFP cells, indicating cells that have lost the switch construct. RAW-togFP cells were first gated on BFP+. Where not seen, error bars (± SEM, standard error of the mean) are smaller than the symbol.

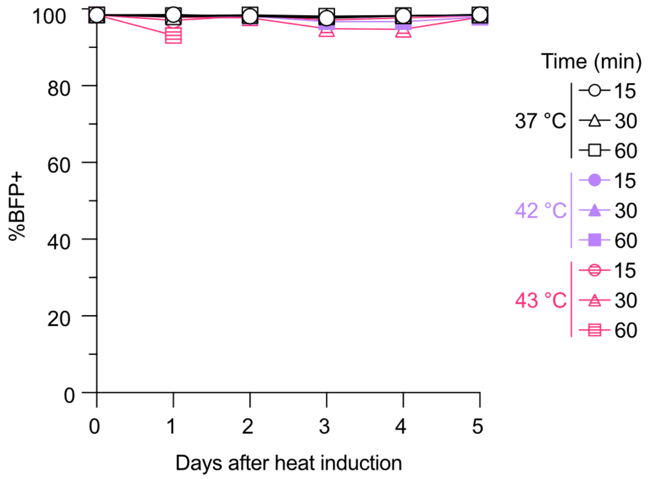

Figure S2. Heat induction does not affect HSP-Cre transgene stability.

Percentage of BFP+ cells, indicating presence of HSP16-Cre construct, remains stable over 5 days following heat induction. Where not seen, error bars (± SEM, standard error of the mean) are smaller than the symbol.

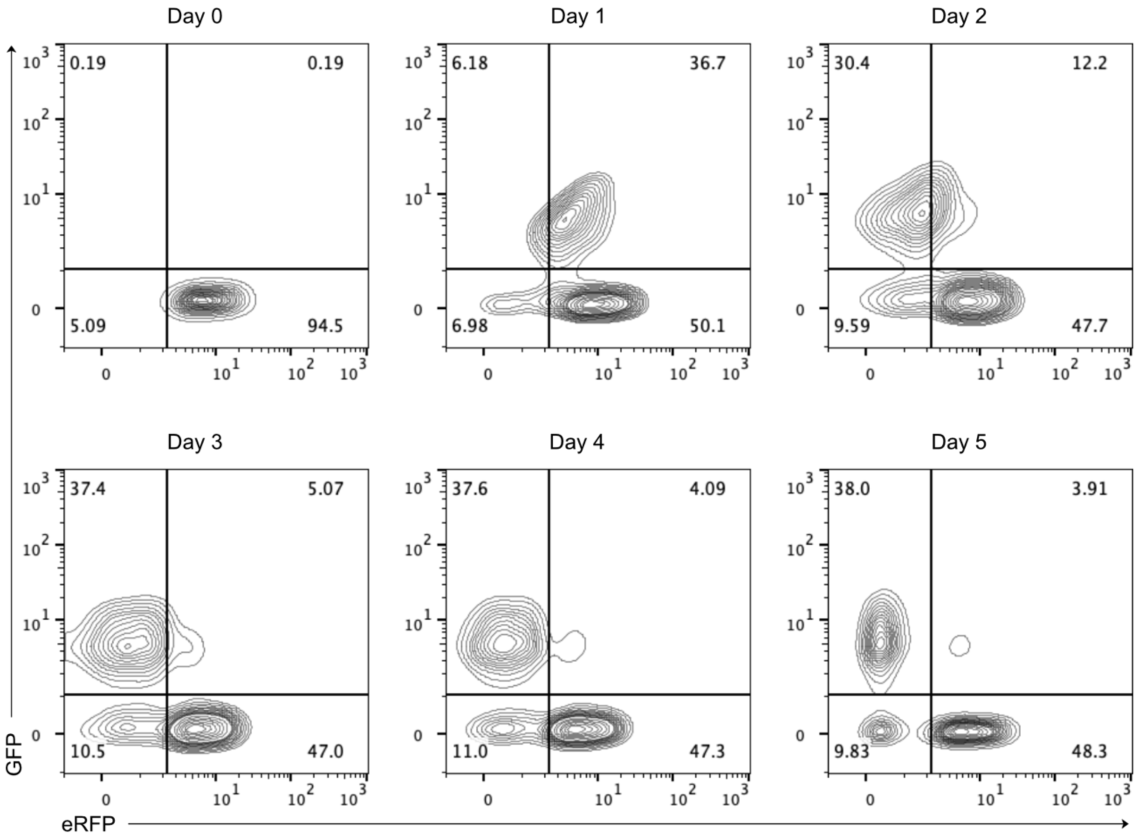

Figure S3. RAW-togFP expression of OFF- and ON-state reporters over five days.

Activated RAW-togFP cells are double-positive (GFP+/eRFP+) one day after heat induction, and gradually shift to GFP+/eRFP− over five days. Cells were first gated for doublet discrimination and BFP+.

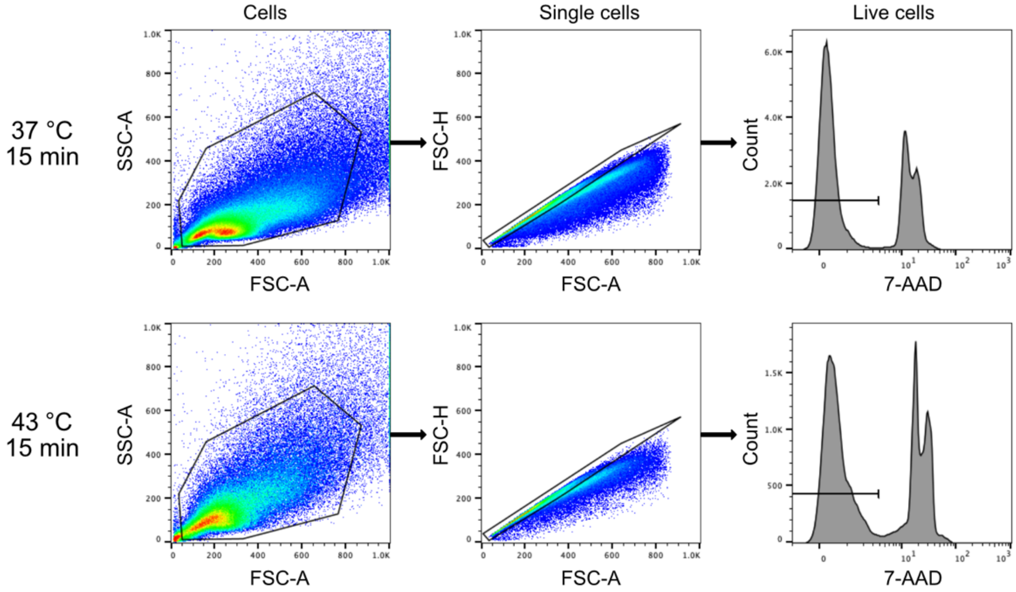

Figure S4. Gating strategy for live/dead staining for counting viable cells.
Cells were first gated on FSC-A vs. SSC-A to exclude debris, then gated on FSC-A vs. FSC-H to exclude doublets. Cells were then gated for 7-AAD exclusion to determine live cell count.

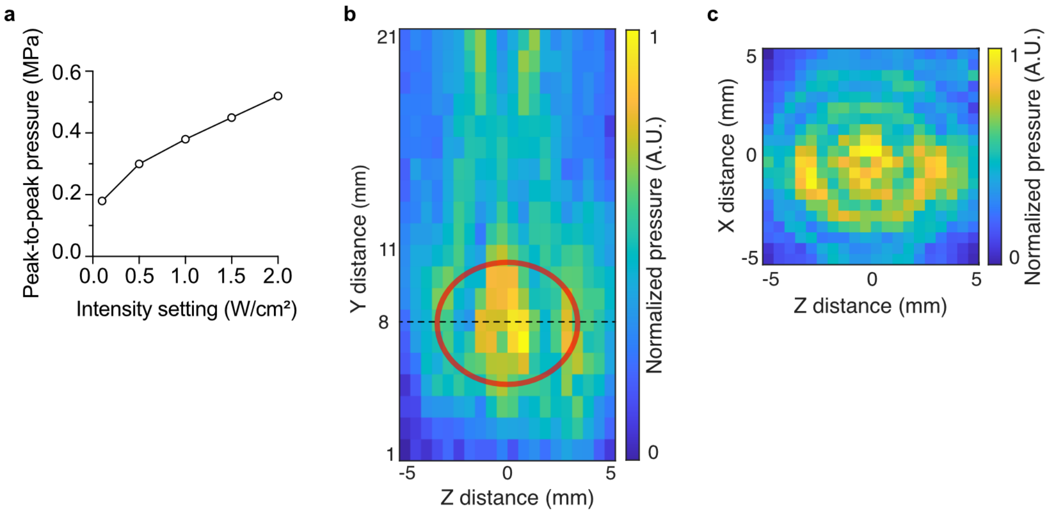

Figure S5. Characterization of the unfocused 3 MHz ultrasound transducer.

a) Calibration plot of peak-to-peak pressure at a single point in the center of the focal region of the transducer under varying applied intensity settings, measured using a needle hydrophone. (b,c) Normalized pressure field of the 3 MHz transducer in the longitudinal plane, along the direction of propagation on the y-axis where the transducer face is positioned at y = 0 (b), and in the transverse plane at y = 8 mm (dashed black line), orthogonal to direction of propagation (c). Red circle represents where the targeted tumor would lie within the longitudinal plane.

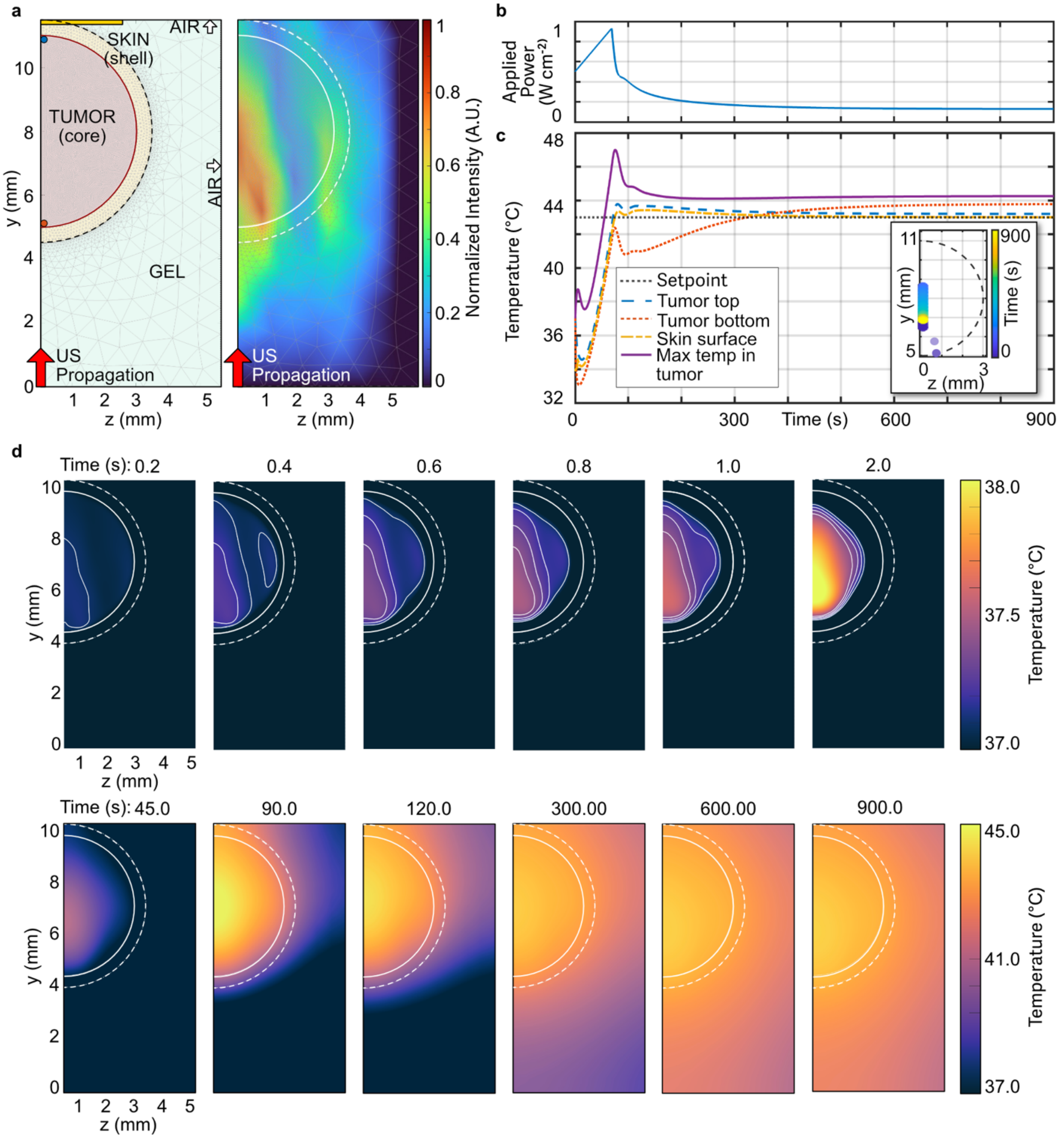

Figure S6. Finite element model simulation of ultrasound-induced intratumoral temperature distribution.

a) Computational domain and acoustic field map used as inputs for thermal modeling. Left: schematic of the axisymmetric domain including gel coupling medium, skin shell, and tumor tissue. Air-facing top and right boundaries used natural convection to ambient $T_\infty = 22$ °C. Symbols indicate regions graphed in panel c (yellow rectangle, skin surface; blue circle, tumor top; red circle, tumor bottom). Right: normalized acoustic intensity field mapped onto the mesh. b) Simulation of PID-controlled applied power, with target temperature at skin surface ROI set to 43 °C. c) Simulated tumor temperature under PID-controlled ultrasound heating at the skin surface (dashed yellow line), top (dashed blue line), bottom

(dashed red line), and maximum temperature within the tumor (solid purple line) over the 15-min heating period. In the initial ~10 seconds, the decrease in temperature can be attributed to heat dissipation into the surrounding medium, prior to sufficient acoustic energy deposition to establish a net positive thermal balance within the tissue. Inset: Location of maximum temperature within the tumor over time (solid purple line). d) Spatial temperature distributions under PID-controlled ultrasound heating at representative time points on the YZ axis. Isothermal lines on top row are drawn at 37.1, 37.2, 37.3, and 37.4 °C.

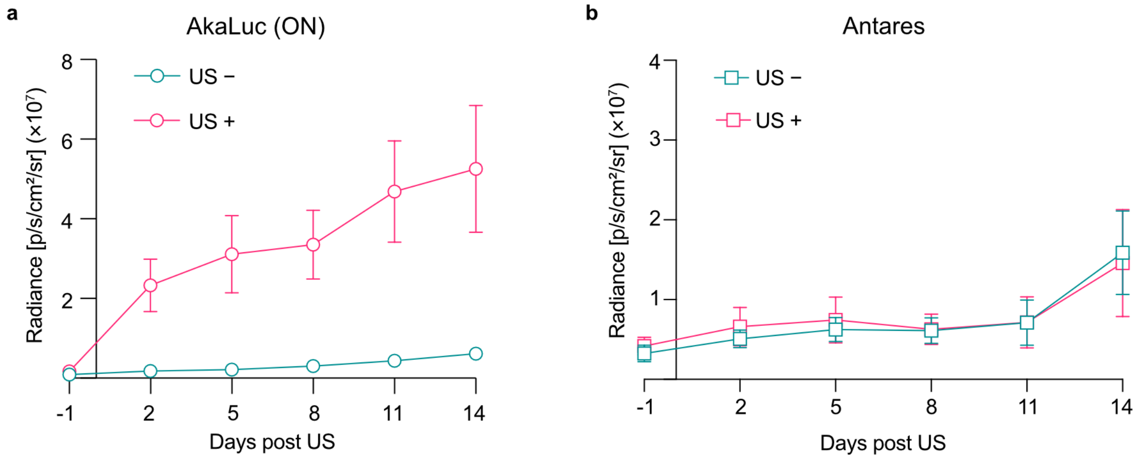

Figure S7. 4T1 + RAW-togLuc tumor growth curves and AkaLuc expression over time.

a) Quantification of AkaLuc (ON) luminescence from tumors treated with US (pink) or untreated on the opposite flank (blue). b) Growth curves of 4T1+RAW-togLuc tumors, measured via quantification of Antares luminescence, treated with US (pink) or untreated on the opposite flank (blue). N = 7 mice. Where not seen, error bars (± SEM, standard error of the mean) are smaller than the symbol.

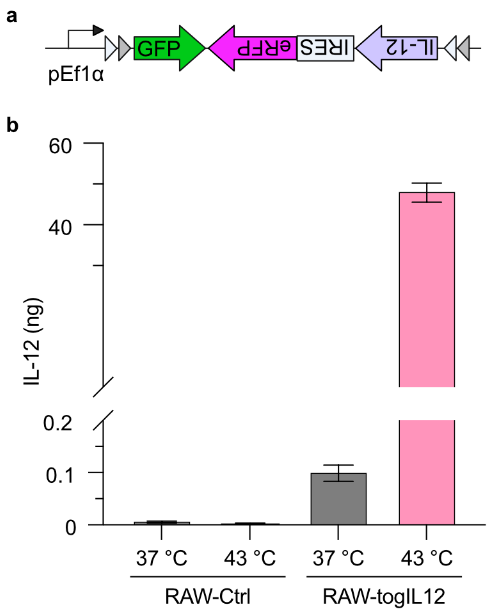

Figure S8. RAW-Ctrl cells do not produce significant amounts of IL-12 after heat induction.

a) RAW cells were transduced with only the toggle switch element of the togIL12 thermal circuit to generate RAW-Ctrl cells. The HSP16-Cre actuator element was not transduced. b) IL-12 production quantified from culture media of RAW-Ctrl and RAW-togIL12 cells three days after incubation at 37 or 43 °C for 15 minutes. n = 3 replicates. Bars = SEM.

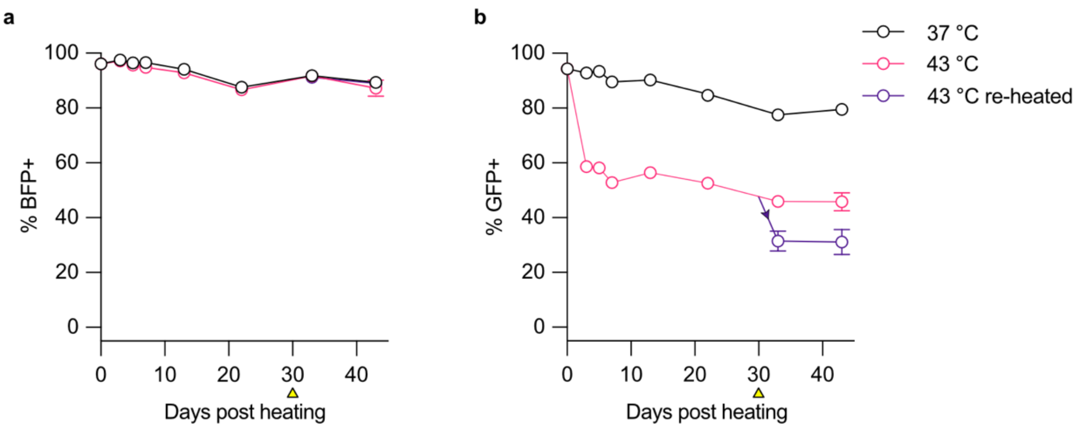

Figure S9. RAW-togIL12 constitutive and OFF-state reporter expression.

RAW-togIL12 cells were incubated at 37 and 43 °C for 15 minutes and then analyzed via flow cytometry for 43 days. Percent BFP+ cells (a) and percent GFP+ of BFP+ cells (b) were determined after first gating for doublet discrimination. At t = 30 days (yellow arrowhead), a subpopulation of cells that had been heated at 43 °C at t = 0 were incubated again at 43 °C for 15 minutes and then further assessed on days 33 and 43 (purple lines). Where not seen, error bars (± SEM, standard error of the mean) are smaller than the symbol.

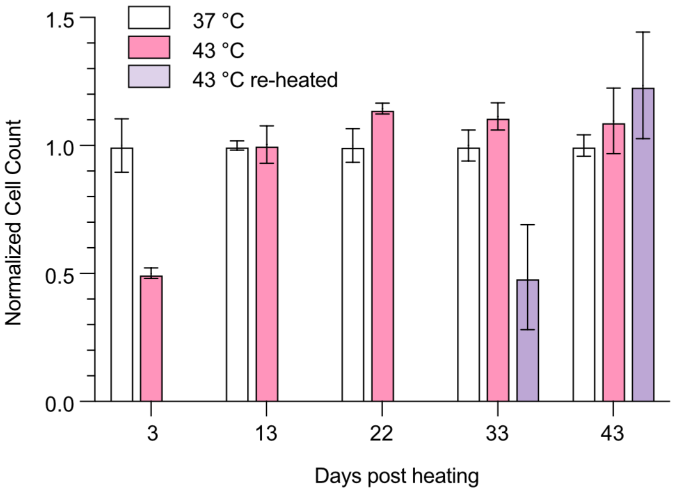

Figure S10. RAW-togIL12 cell proliferation over 43 days.

Cell count of RAW-togIL12 cells incubated at 37 and 43 °C for 15 minutes on day 0 normalized to the 37 °C condition. After treatment, cells were plated, then collected on day 3 and counted using flow cytometry. For each subsequent time point, an equal number of heated and unheated cells were seeded three days prior to collection. Cell proliferation was assessed by the cell count after 3 days of culture. On day 30 after heating, a subpopulation of cells that had been heated at 43 °C on day 0 were incubated again at 43 °C for 15 minutes and then counted on days 33 and 43.

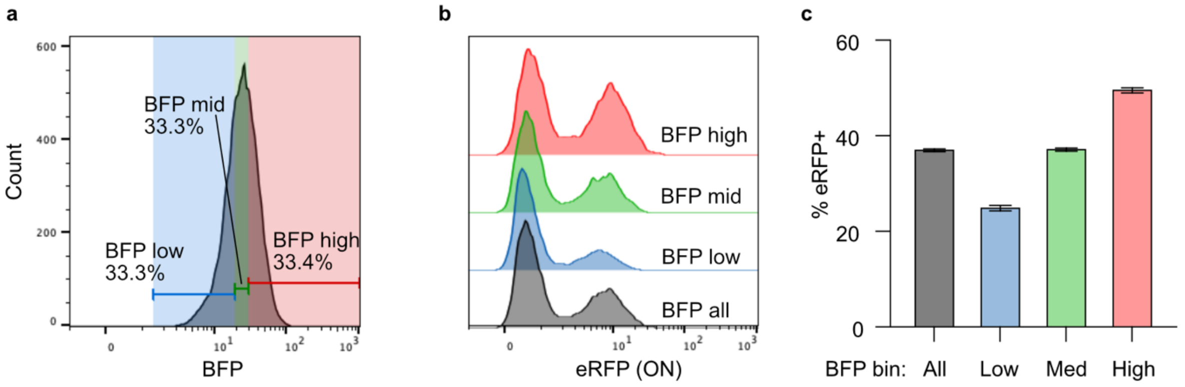

Figure S11. togIL12 circuit activation after binning on BFP expression in RAW-togIL12 cells.
RAW-togIL12 cells were heated at 43 °C for 15 minutes and then analyzed via flow cytometry. a) BFP+ cells were gated into the lowest 33% (BFP low), median 33% (BFP mid), and highest 33% (BFP high) bins of BFP expression. b-c) Each BFP expression bin was then analyzed for eRFP (ON state) expression.

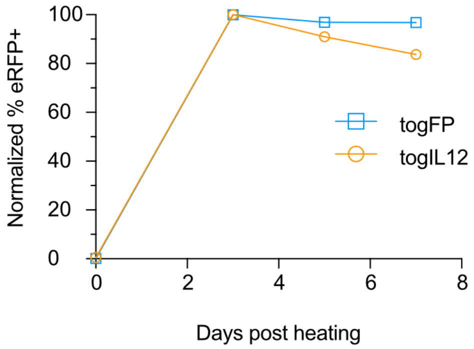

Figure S12. Difference in “ON state” switch loss between togFP and togIL12 constructs. Percent eRFP+ (activated) normalized to the peak value for RAW-togFP and RAW-togIL12 cells after heat induction at 43 °C for 15 minutes. Cells were first gated on BFP (marker for the HSP-Cre construct).
